# Identification and Application of Two Promising Peptide Ligands for the Immunodetection of Imidacloprid Residue

**DOI:** 10.3390/foods11203163

**Published:** 2022-10-11

**Authors:** Tianyang You, Yuan Ding, Yue Huang, Yang Lu, Minghua Wang, Xiude Hua

**Affiliations:** 1 College of Plant Protection, Nanjing Agricultural University, Nanjing 210095, China; 2 State & Local Joint Engineering Research Center of Green Pesticide Invention and Application, Nanjing 210095, China

**Keywords:** imidacloprid, anti-immunocomplex peptide, peptidomimetic, noncompetitive immunoassay, competitive immunoassay

## Abstract

As the most widely used neonicotinoid insecticide, it is of great significance to explore the immunoreagents and immunoassays for imidacloprid (IMI) residue. In immunoassays, specific peptide ligands, such as peptidomimetic and anti-immunocomplex peptides, are regarded as promising substitutes for chemical haptens. In the present work, we identified thirty sequences of peptidomimetics and two sequences of anti-immunocomplex peptides for IMI from three phage pVIII display cyclic peptide libraries, in which the anti-immunocomplex peptides are the first reported noncompetitive reagents for IMI. The peptidomimetic 1-9-H and anti-immunocomplex peptide 2-1-H that showed the best sensitivity were utilized to develop competitive and noncompetitive phage enzyme-linked immunosorbent assays (P-ELISAs), with a half inhibition concentration of 0.55 ng/mL for competitive P-ELISA and a half-saturation concentration of 0.35 ng/mL for noncompetitive P-ELISA. The anti-immunocomplex peptide was demonstrated to greatly improve the specificity compared with competitive P-ELISA. In addition, the accuracy of proposed P-ELISAs was confirmed by recovery analysis and HPLC verification in agricultural and environmental samples. These results show that the peptide ligands identified from phage display library can replace chemical haptens in the immunoassays of IMI with the satisfactory performance.

## 1. Introduction

Immunoassay plays an increasingly important role in pesticide residue detection. Although the precision and reliability of chromatography-based methods are apparent, the advantages of immunoassay, such as its low-cost, simple operation and portable detection device, make it the preferable approach for screening large samples and for on-site detection. The establishment of immunoassay is realized by the recognition between immunorecognition reagents (antibody and antigen) [1,2]. The chemical hapten and monoclonal antibody (mAb) are the classic combination of immunorecognition reagents, which has achieved great success in the immunoassays for pesticide residue [3,4]. Even though there is still no clear design rule for high quality hapten, trial and error are the commonly used methods [5,6,7]. Given the great variety of pesticides, preparation of high quality hapten for each pesticide means a tremendous amount of work. Besides, the competitive immunoassay based on chemical hapten generates a negative correlation signal that makes the detection results not intuitive.

In recent years, the specific peptide screened from the phage display peptide library has shown the potential to replace chemical hapten as the next-generation immunoreagent in pesticide residue immunoassay [8]. The peptides can work in two different ways. One way is the same as with chemical haptens, which directly reacts with the antibody (called peptidomimetic) [9]. The other way is more special, in which the peptide reacts with the immunocomplex between antibody and antigen (called as anti-immunocomplex peptide) to establish the noncompetitive immunoassay [10]. The noncompetitive format provides a positive correlation signal that makes the detection results more friendly. Unlike open sandwich immunoassays or the noncompetitive immunoassay based on an anti-idiotype antibody, anti-immunocomplex peptide is easier to obtain so that it is increasingly being reported in pesticide residue immunoassay [11,12,13,14]. These two types of peptides generally improved the sensitivity or specificity of immunoassays in previous reports. More importantly, the screening procedure can be completed within a week in a basic biology laboratory, which is of great significance to preparing the immunoreagent of various pesticides.

Imidacloprid (IMI) is the first registered and most widely used neonicotinoid. It acts on the nicotinic acetylcholine receptors of pests so that it disturbs the normal nerve conduction, it is mainly used against Homoptera, Thysanoptera, Coleoptera, Diptera and Lepidoptera pests. Although IMI has been completely banned from outdoor use by the European Commission (EU) to protect honeybees, it is still registered and used globally in crop and ornamentals protection, urban pest control, veterinary applications, and fish farming, owing to its excellent systemic and contact activity. The widespread use and persistence property render IMI detectable in all kinds of agricultural, water, and soil samples worldwide [15,16,17]. Besides the high toxicity to honeybees, there is some research which showed that IMI exposure was positively correlated with several diseases in newborns [18,19,20] and oxidative DNA damage in adults [21,22,23]. Governments regulate the maximum residue limits (MRLs) of imidacloprid in various food. China has regulated the MRLs of imidacloprid in 123 kinds of foods, which range from 0.01 mg kg^−1^ to 10 mg kg^−1^ (GB2763-2021). The EU has regulated the MRLs of imidacloprid in 381 kinds of food with the range of 0.01 mg kg^−1^ to 15 mg kg^−1^ (EU 2021/1881). The U.S. Environmental Protection Agency has regulated the MRLs of imidacloprid in 134 kinds of food with the range of 0.02 mg kg^−1^ to 240 mg kg^−1^. Even though IMI has been banned from outdoor use in EU, it is still necessary to monitor IMIs concentration in the environment. So, it is desirable to develop the analytical method for imidacloprid residue. Currently, dozens of immunoassays for IMI residue have been reported, most of which are competitive immunoassays based on chemical haptens [24,25,26,27,28,29], but there is no reported noncompetitive immunoreagent for IMI.

In our recent study, we optimized the pVIII phage display peptide system to improve the panning success rate [13]. In the present work, IMI was determined as a target and an anti-IMI mAb 3D11B12E5 was employed to screen above the pVIII phage display peptide libraries. Then, peptidomimetic and anti-immunocomplex peptides were used to develop competitive and noncompetitive phage enzyme-linked immunosorbent assays (P-ELISAs) for IMI, and then the sensitivities and specificities of the P-ELISAs were compared. Finally, the competitive and noncompetitive P-ELISAs were utilized to test the IMI spiked samples, which the results were verified with high performance liquid chromatography (HPLC).

## 2. Materials and Methods

### 2.1. Reagents

Imidacloprid (99.5%) and imidaclothiz (97.8%) were obtained from Dr. Ehrenstorfer (Augsburg, Germany) and from Jiangshan Agrochemical and Chemicals Co., Ltd. (Nantong, China), respectively. Other compounds for specificity test were all purchased from Dr. Ehrenstorfer (Augsburg, Germany). The ER 2738 and helper phage M13KO7 were purchased from New England Biolabs (Ipswich, UK). Tween-20 was purchased from Solarbio (Beijing, China). Skimmed milk was purchased from Bio-Rad (Hercules, CA, USA). The HRP labelled anti-M13 antibody was purchased from SinoBiological (Beijing, China). The ninety-six-well microplates were purchased from Corning Costar (Corning, NY, USA). The mAb 3D11B12E5 purified by protein A column and phage-displayed cyclic 8, 9, 10-residue random peptide libraries were prepared and stored in our own laboratory [13,30]. The sequencing primer was synthesized by Genscript Bio. (Nanjing, China).

### 2.2. Biopanning

Anti-IMI mAb 3D11B12E5 diluted in PBS was added to nine wells of microplate at 4 °C overnight (100 μL/well). The plate was washed three times by PBST (PBS containing tween-20) and blocked with 300 μL 5% skimmed milk (dissolved in PBS) at 37 °C for 2 h. For peptidomimetic panning, 100 μL/well mixture of phage-displayed cyclic 8-, 9-, 10-residue random peptide libraries was diluted using 5% skimmed milk, and added to three coated wells, and incubated at room temperature for 1 h. The plate was washed ten times with PBST, incubated with PBS for 30 min, and then washed ten times with PBS. After washing, 100 μL/well IMI standard solution was added and incubated at room temperature for 1 h to competitively elute the bound phage. For immunocomplex panning, 100 μL/well IMI standard solution (10 μg/mL) was firstly injected to form immunocomplex for 1 h. After discarding the excess IMI, the mixture of phage-displayed peptide libraries used in peptidomimetic panning was added and incubated at room temperature for another 1 h. After washing, the bound phage was eluted by an elution buffer (0.2 M Gly-HCl containing 1 mg/mL BSA, pH 2.2) for 15 min, and immediately neutralized to pH 7.4 using 1 M Tris-HCl (pH 9.1). The eluted phages were titer-determined and amplified [13] for subsequent panning.

A total of three rounds of panning were performed under different panning conditions. The concentrations of PBST used in the first, second, and third rounds were 0.1%, 0.3%, and 0.5%. The coating concentrations of mAb were 10, 5, and 2.5 μg/mL for the first, second, and third rounds. The quantities of phage inputted in the first, second, and third rounds of panning were 3 × 10^11^, 3 × 10^10^ and 3 × 10^10^ pfu. The concentrations of IMI standard solution in peptidomimetic panning were 10, 5 and 2.5 μg/mL. After three rounds of panning, a total of 32 clones from the last titer-determining plate were picked and amplified for the identification of positive clones using P-ELISAs.

### 2.3. P-ELISAs

100 μL/well mAb 3D11B12E5 diluted in PBS was coated in the microplate at 4 °C overnight. After being washed three times with 0.5‰ P BST, the plate was blocked with 300 μL/well 5% skimmed milk (diluted in PBS) at 37 °C for 1.5 h. After washing, fifty microliters of IMI standard or sample solution and fifty microliters of phage-displayed peptide diluted using 5% skimmed milk was added to the plate, and reacted at 37 °C for 1 h. After washing, 100 μL anti-M13 antibody (HRP) diluted with 0.05% PBST (0.2 μg/mL, containing 0.5% skimmed milk) was added to the recognized phage particles. After washing, 100 μL of peroxidase substrate (10 mL 0.1 M citric acid and dibasic sodium phosphate buffer (pH 5.5), 32 μL 0.75% H_2_O_2_, and 100 μL 10 mg/mL TMB in dimethyl sulfoxide) was added and reacted at 37 °C for 15 min, then stopped with 50 μL 2 M H_2_SO_4_. The optical density at 450 nm (OD_450_) was determined with a SpectraMax M5 microplate reader. The dose-response curve was fitted by logistic equation using the IMI concentration on the *X* axis and OD_450_ value on the *Y* axis.

### 2.4. Optimization of P-ELISAs

The optimal concentrations of mAb 3D11B12E5 and phage-displayed peptides were determined by the checkboard method, in which a series of concentrations of mAb 3D11B12E5 were combined with various quantities of phage-displayed peptides to run P-ELISAs, respectively. For determining the optimal buffer, the dose-response curves of P-ELISAs were created in various PBS buffers, which contained different Na^+^ concentrations (from 0.035 to 2.4 mol/L), pH values (from 5.0 to 9.0), and methanol contents (from 1.25% to 10%).

### 2.5. Specificity

The specificities of P-ELISAs, defined as the cross-reactivities (CRs) with other analogs of IMI, were calculated as follows: CR = [IC_50_ (or SC_50_) _IMI_/IC_50_ (or SC_50_) _analogs_] × 100%.

### 2.6. Analysis of Spiked Samples

The accuracies of proposed P-ELISAs for IMI detection in various agricultural (wheat, brown rice, potato, cabbage, pear, and orange) and environmental samples (paddy water and soil) were evaluated. The samples were collected from Nanjing, China. The agricultural samples were chopped and homogenized. The paddy water samples were filtered, while the soil samples were air-dried, crushed, and passed through a 2 mm sieve. All of the samples were stored at −20 °C. The samples were confirmed not to be contaminated with IMI by HPLC.

The samples were spiked with various concentrations of IMI standard solutions and stood overnight. Before the analysis of the spiked samples, their matrix effect on P-ELISAs were firstly investigated by preparing the dose-response curves in series diluted blank extracts, then compared with the PBS-based standard curve to determine the dilution factors. After dilution, the paddy water was directly detected without further processing. For other solid samples, 5 g samples were added to a 50 mL centrifuge tube, then extracted by 10 mL optimized PBS containing 45% methanol. The tube was vortexed at 300 rpm for 5 min, sonicated for 15 min, then vortexed for another 5 min. After centrifuging at 4000 rpm for 5 min, the extracts were collected, and the extracts of cabbage, pear, potato, and orange were adjusted to 15 mL by PBS. The extracts of spiked samples were tested by P-ELISAs after proper dilutions.

### 2.7. Verification of P-ELISAs by HPLC

IMI standard solutions with unknown concentrations were spiked into pear and soil samples, respectively. For P-ELISAs, the samples were processed and analyzed as above. For HPLC, the samples were processed and analyzed as previously described [9].

## 3. Results

### 3.1. Panning Results

The peptide libraries used in this study are displayed on phage pVIII in high-density, whose expressions are controlled by the *lac* promoter/operator. Such a display strategy may increase the apparent affinity and weaken the growth bias among different phages, thus serving as a footstone for the blended panning strategy using mixed libraries [13].

**Figure 1:**
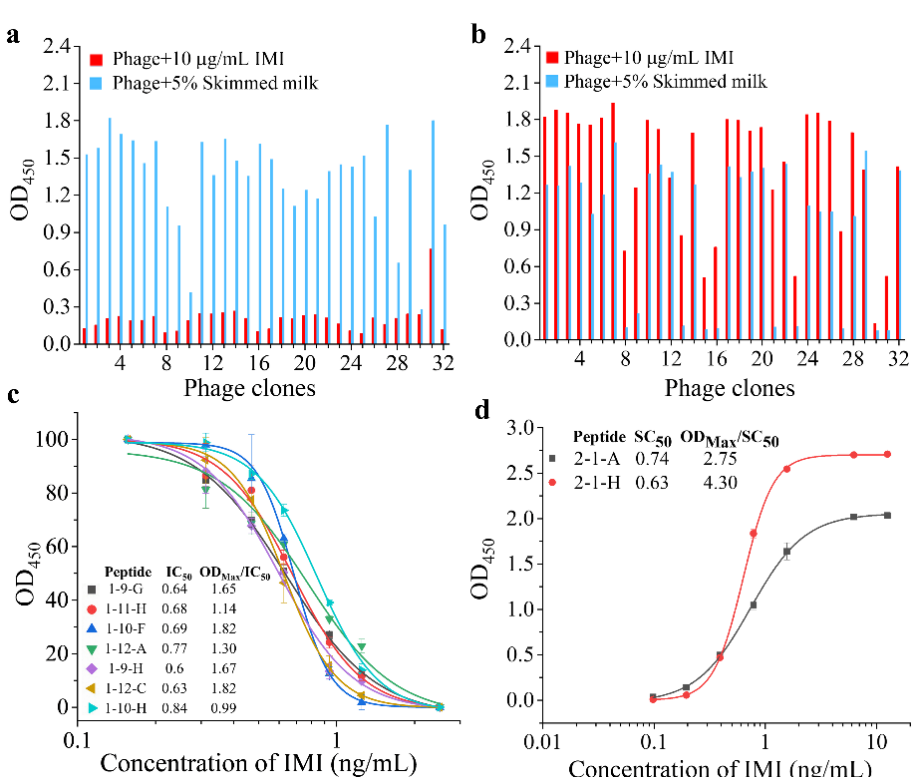
Panning results. (**a**) The screen of peptidomimetics. (**b**) The screen of anti-immunocomplex peptides. (**c**) Dose-response curve of different peptidomimetics. (**d**) Dose-response curve of different anti-immunocomplex peptides.

The phage titer of each panning is shown in Table S1. While the screening condition got more stringent, the titers showed an increase of 100–1000 fold after the third round of panning, indicating an enrichment of desired peptidomimetics or anti-immunocomplex peptides. After identification, thirty clones showed decreased signals in the presence of IMI (Figure 1a), and twenty eight clones showed enhanced signals in the presence of IMI (Figure 1b). The sequences of these positive clones are shown in Table 1, in which a total of thirty sequences of peptidomimetics and two sequences of anti-immunocomplex peptides are identified. Most sequences of peptidomimetics contained the consensus motif TPAG, while there is no consensus motif among the anti-immunocomplex peptides. While the 8-, 9-, 10-residue peptide libraries were mixed equally for panning, peptides from the 9-residue peptide library were not included in the anti-immunocomplex peptides. It’s a common fact that peptides bound to the immunocomplex of a given target may not exist in one particular library. Panning through multiple libraries is necessary to increase the chance, and a blended manner will help reduce the heavy workload.

### 3.2. Optimization of P-ELISAs

The sensitivities of seven peptidomimetics (1-9-G, 1-9-H, 1-10-F, 1-10-H, 1-11-H, 1-12-A, 1-12-C) that showed enhanced signal differences in the presence of IMI, and all anti-immunocomplex peptides (2-1-A, 2-1-H), were evaluated by P-ELISAs. The optimal concentration of mAb and input quantities of phage-displayed peptides were firstly determined. The dosages of mAb 3D11B12E5 and phage-displayed peptides that generated the OD_450_ ≈ 1 were chosen to develop dose-response curve for competitive P-ELISA (Table S2 and Figure 1c). The dosages that produced the highest S/N were chosen to develop dose-response curves for noncompetitive P-ELISA (Figure S1 and Figure 1d). The peptidomimetics 1-9-H showed the lowest IC_50_ and the highest maximum OD_450_ (OD_max_)/IC_50_ were selected for subsequent experiments, while the anti-immunocomplex peptides 2-1-H were selected.

**Figure 2:**
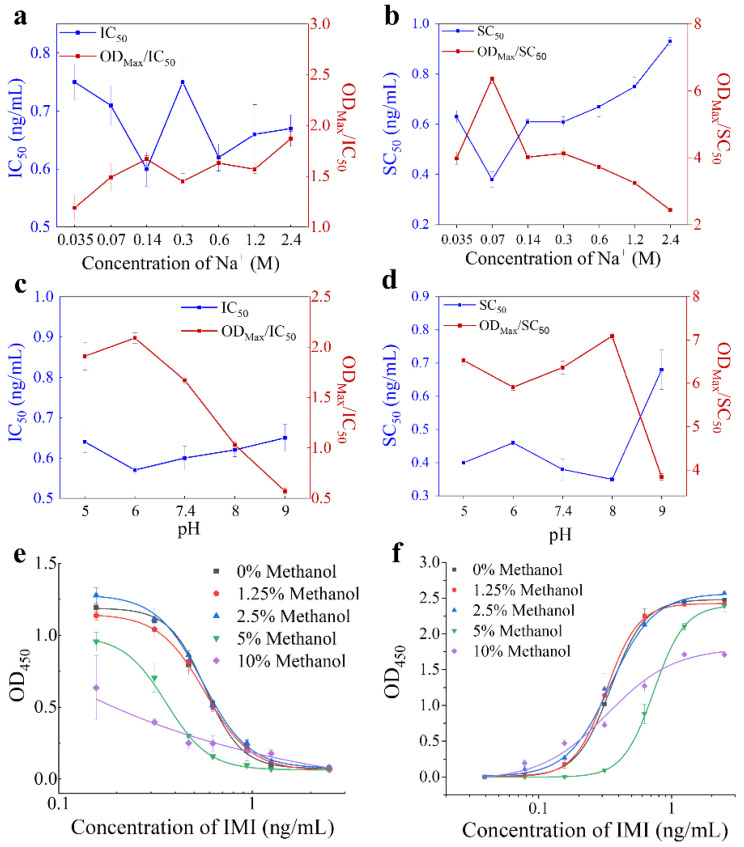
Optimization of P-ELISAs. (**a**,**c**,**e**) The concentration of Na^+^, pH and methanol for competitive P-ELISA. (**b**,**d**,**f**) The concentration of Na^+^, pH and methanol for noncompetitive P-ELISA.

The binding between antigen and antibody is mediated by Van der Waals, hydrogen and ion force, which will be influenced by the pH and ionic strength in reaction environment [31]. As an effective solvent for pesticide extraction, methanol is also inevitable in their immunoassays. To achieve the best sensitivity, these conditions in P-ELISAs were subsequently optimized. The conditions that generate the dose-response curve with the lowest IC_50_ (or SC_50_) and the highest OD_max_/IC_50_ (or OD_max_/SC_50_) were considered as the optimums. As shown in Figure 2, the PBS buffer containing 0.14 M Na^+^ and pH 6.0 was chosen to develop competitive P-ELISA, and the PBS buffer containing 0.07 M Na^+^ and pH 8.0 was chosen to develop noncompetitive P-ELISA. Reaction between the antibody and phage display peptides was significantly disturbed when the methanol concentrations in the buffer were higher than 2.5% (Figure 2e,f), so the maximum tolerance of P-ELISAs to methanol was 2.5%. In order to avoid the interference caused by different methanol concentrations in the pretreatment of various samples, the methanol concentrations were all determined as 2.5%.

### 3.3. Sensitivity of P-ELISAs

**Figure 3:**
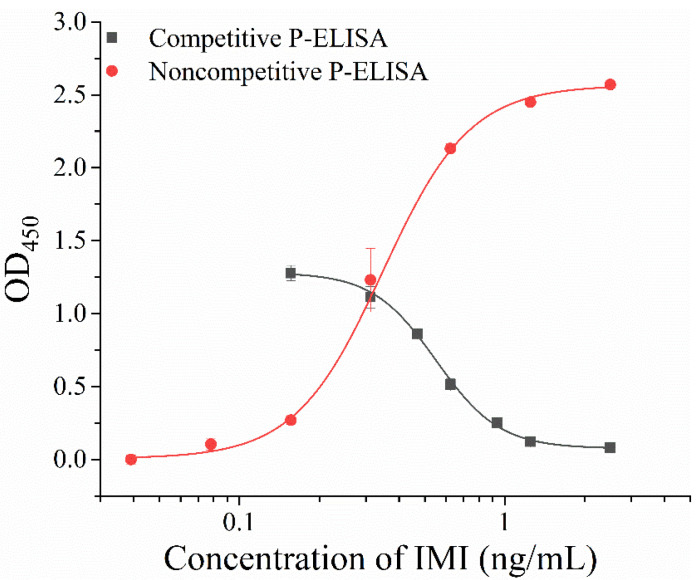
Standard curve of P-ELISAs.

The dose-response curves of two P-ELISAs are shown in Figure 3. The IC_50_ of competitive P-ELISA was 0.55 ng/mL, with the limit of detection (LOD, IC_10_) of 0.30 ng/mL and detection range (IC_10_–IC_90_) of 0.30–1.00 ng/mL, while the SC_50_ of noncompetitive P-ELISA was 0.35 ng/mL, with the LOD of 0.15 ng/mL and detection range (SC_10_–SC_90_) of 0.15–0.80 ng/mL. The noncompetitive P-ELISA showed slightly improved sensitivity than competitive P-ELISA. We have summarized the reported sensitivities of the basic immunodetection, that is ELISA, for the detection of IMI (Table S3). Except for the P-ELISAs reported here, there are two other competitive P-ELISAs using phage displayed peptidomimetics [9,32] and six competitive ELISAs using chemical haptens [24,25,26,27,28,29]. The proposed P-ELISAs showed better sensitivities than most reported ELISAs, just lower than another competitive P-ELISA using a pIII displayed linear 12-amino acid peptidomimetic. The latter immunoassay, which was reported by Du et al. [9], achieves the highest sensitivity (IC_50_ = 0.067 ng/mL). The HRP labelled anti-M13 antibody used in this work is targeted at phage pVIII, whose binding may be hampered by the phage pVIII displayed peptides, thus causing a lower signal amplification effect and leading to the poor sensitivity of P-ELISAs. It is worth mentioning that isolated peptides here are cyclic peptides, which may mean better stability. Moreover, the noncompetitive immunoreagent for imidacloprid is reported for the first time, which can derive a series of noncompetitive immunoassays for imidacloprid with more intuitive detection results. It has advantages in the development of visual detection.

### 3.4. Specificity of P-ELISAs

The specificity of P-ELISAs, defined as CR, is shown in Table 2. Due to the similar structure of neonicotinoids, the competitive P-ELISA has 105.8%, 70.5%, 27.1%, 14.8% and 11.6% CRs to imidaclothiz, clothianidin, thiacloprid, nitenpyram and acetamiprid. Meanwhile, thanks to the two-site recognition pattern of anti-immunocomplex peptides, the noncompetitive P-ELISA greatly reduces the CRs of clothianidin, thiacloprid, nitenpyram and acetamiprid to <0.1%, 21.7%, and <0.1% and 6.8%. The CRs to imidaclothiz is a widespread problem in the immunoassays for imidacloprid [33]. Regrettably, the utilization of anti-immunocomplex peptides has not solved this problem either.

### 3.5. Recoveries of Spiked Samples

The matrix effect that would cause the false result has to be solved before testing actual samples. Owing to the high sensitivity of immunoassays, the matrix effects of extracts can simply be eliminated by the dilution method. To evaluate their influence, series diluted matrix extracts were used to replace the optimal buffer and run the dose-response curves of P-ELISAs. When the dilution fold produces a similar curve to the standard curve, the matrix effect is considered to be eliminated. As shown in Figures S2 and S3, the effect of matrix extracts on competitive P-ELISA can be eliminated after 2-fold dilution of paddy water, total 36-fold dilution of soil and pear, 72-fold dilution of orange and brown rice, and 144-fold dilution of cabbage, potato, and wheat, while the effect on noncompetitive P-ELISA can be eliminated after 2-fold dilution of paddy water, 36-fold dilution of potato and orange, 72-fold dilution of soil, wheat, cabbage, and pear, and 144-fold dilution of brown rice.

After the elimination of matrix effects, the IMI concentrations in the spiked samples were determined (Table 3). The average recoveries of competitive and noncompetitive P-ELISAs were 75.1–103.5% and 73.8–101.0%, with relative strand deviations (RSDs) of 1.7–7.8% and 2.0–9.3%, respectively. The results agreed with the IUPAC standard [34] regulation, “average recoveries among 70–120%, RSD ≤ 20%”.

### 3.6. Verification by HPLC

**Figure 4:**
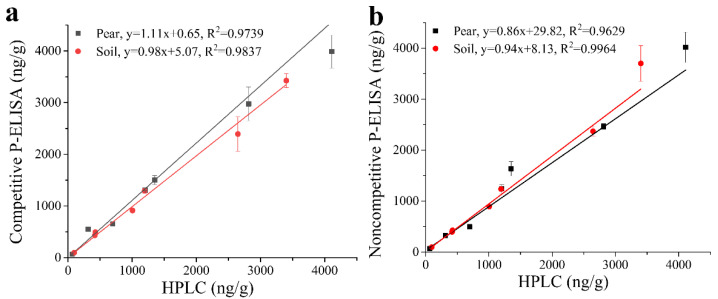
Blind samples tested by P-ELISAs and HPLC. (**a**) Competitive P-ELISA. (**b**) Noncompetitive P-ELISA.

As a gold standard for pesticide residue detection, HPLC was used to further verify the accuracy of the proposed P-ELISAs. The blind samples of pear and soil were simultaneously detected by HPLC and the P-ELISAs. The results are shown in Figure 4. The correlation equations between competitive P-ELISA and HPLC are y = 1.11x + 0.65 (R^2^ = 0.9739) for pear and y = 0.98x + 5.07 (R^2^ = 0.9837) for soil, while y = 0.86x + 29.82 (R^2^ = 0.9629) for pear and y = 0.94x + 8.13 (R^2^ = 0.9964) for soil between noncompetitive P-ELISA and HPLC. The slope and R^2^ of the equations are close to 1, which indicates a good correlation between HPLC and the immunoassays.

## 4. Conclusions

In this study, thirty sequences of peptidomimetics and two sequences of anti-immunocomplex peptides for IMI were isolated from phage display peptide libraries, among which the anti-immunocomplex peptides were the first reported noncompetitive reagent for IMI. The peptidomimetic 1-9-H and anti-immunocomplex peptide 2-1-H that showed the best sensitivities were utilized to develop competitive and noncompetitive P-ELISAs. The IC_50_ and LOD of competitive P-ELISA were 0.55 ng/mL and 0.3 ng/mL, compared with 0.35 ng/mL and 0.15 ng/mL for noncompetitive P-ELISA. The sensitivities of P-ELISAs were better than most reported immunoassays for IMI, which can be further improved by collaborating with other tracers. Moreover, the two-site recognition pattern of anti-immune complex peptides in noncompetitive P-ELISA greatly improved the specificity compared with competitive P-ELISA. As confirmed by the recoveries analysis and HPLC verification, the proposed P-ELISAs can detect IMI in agricultural and environmental samples accurately and reliably. IMI residue has always been the focus of attention due to IMIs wide use. So, we believe that the specific peptide sequences and methods reported here are helpful to detect IMI residue. Further, unlike chemical hapten, peptide ligands can be prepared using a biological expression, so it is attractive to explore the peptide ligands to derive novel patterns of immunoassay, such as label-free immunoassay.

## Figures and Tables

**Table 1:** Sequence of isolated peptidomimetics and anti-immunocomplex peptides.

Clone	Amino Acid Quantity	Amino Acid Sequence	Clone	Amino Acid Quantity	Amino Acid Sequence
Peptidomimetics (consensus TPAG)					
1-9-A	8	CFE**TPAG**LMC	1-11-A	8	CTMPD**PAG**MC
1-9-B	8	CEM**TPAG**WLC	1-11-B	8	CLE**TPAG**LAC
1-9-C	10	MS**PAG**IWDAQC	1-11-C	8	CVAS**PAG**LVC
1-9-D	8	CES**TPAG**YFC	1-11-D	8	CAA**TPAG**LVC
1-9-E	9	CTD**PAG**MLASC	1-11-E	8	CHSS**PAG**FVC
1-9-F	9	CTF**TP**SVRRIC	1-11-F	8	CVE**TPAG**FYC
1-9-G	8	CSMS**PAG**PIC	1-11-G	8	CEW**TPAG**WVC
1-9-H	8	CVP**TPAG**DFC	1-11-H	8	CVD**TPAG**LYC
1-10-A	8	CVS**TPAG**LTC	1-12-A	8	CPL**TPAG**PVC
1-10-C	8	CVMS**PAG**PVC	1-12-B	8	CEM**TPAG**LAC
1-10-D	8	CVSS**P**G**G**LVC	1-12-C	8	CEM**TPAG**PVC
1-10-E	8	CEQ**TPAG**LVC	1-12-D	9	CEMN**PAG**IRIC
1-10-F	8	CEYS**PAG**VIC	1-12-E	8	CEDS**PAG**WIC
1-10-G	8	CLM**TPAG**PSC	1-12-G	8	CHES**P**G**G**MIC
1-10-H	8	CEQ**TPAG**LMC	1-12-H	8	CTMS**P**G**G**WIC
Anti-immunocomplex peptides (No consensus sequence)					
2-1-A	8	CWCIEDCSNC (18) ^1^	2-1-H	10	CVWDGDVGIMYC (9) ^1^

^1^ Quantity of same sequence clones.

**Table 2:** CR of P-ELISAs with other neonicotinoids (*n* = 3).

Compound	Chemical Structure	Competitive P-ELISA		Noncompetitive P-ELISA	
		IC_50_ (ng/mL)	CR (%)	SC_50_ (ng/mL)	CR (%)
Imidacloprid	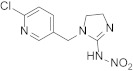	0.55	100.0	0.35	100.0
Imidaclothiz	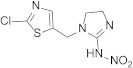	0.52	105.8	0.31	112.9
Thiacloprid	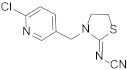	2.03	27.1	1.61	21.7
Acetamiprid	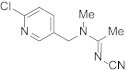	4.75	11.6	5.15	6.8
Clothianidin	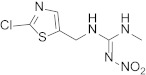	0.78	70.5	>10,000.00	<0.1
Nitenpyram	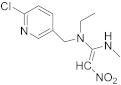	3.71	14.8	>10,000.00	<0.1
Thiamethoxam	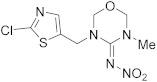	>10,000.00	<0.1	>10,000.00	<0.1
Dinotefuran	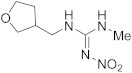	>10,000.00	<0.1	>10,000.00	<0.1

**Table 3:** Recoveries of spiked samples for IMI (*n* = 3).

Matrix	Spiked (ng/g)	Competitive P-ELISA			Noncompetitive P-ELISA		
		Measured ± SD (ng/g)	Average Recovery (%)	RSD (%)	Measured ± SD (ng/g)	Average Recovery (%)	RSD (%)
Paddy water	2	1.81 ± 0.07	90.7	4.1	1.77 ± 0.07	88.5	4.1
	4	3.69 ± 0.10	92.3	2.8	3.56 ± 0.23	88.9	6.4
	8	8.27 ± 0.49	103.4	5.9	7.78 ± 0.41	97.2	5.3
Soil	50	42.81 ± 0.95	85.6	2.2	44.91 ± 2.51	89.8	5.6
	100	90.02 ± 2.66	90.0	3.0	101.01 ± 4.01	101.0	4.0
	200	185.63 ± 4.11	92.8	2.2	186.59 ± 11.78	93.3	6.3
Wheat	50	38.29 ± 2.28	76.6	6.0	42.34 ± 3.41	84.7	8.1
	100	81.17 ± 1.42	81.2	1.7	84.52 ± 7.89	84.5	9.3
	200	181.40 ± 6.84	90.7	3.8	165.99 ± 11.78	83.0	7.1
Brown rice	50	42.04 ± 2.40	84.1	5.7	45.39 ± 1.92	90.8	4.2
	100	85.22 ± 6.64	85.2	7.8	89.86 ± 8.17	89.9	9.1
	200	173.29 ± 12.48	86.6	7.2	192.07 ± 6.66	96.0	3.5
Cabbage	50	44.94 ± 1.89	89.9	4.2	40.47 ± 2.45	80.9	6.0
	100	84.36 ± 3.23	84.4	3.8	81.74 ± 4.04	81.7	4.9
	200	164.56 ± 8.82	82.3	5.4	183.67 ± 5.59	91.8	3.0
Potato	50	44.2 ± 3.04	88.4	6.9	46.79 ± 2.00	93.6	4.3
	100	88.15 ± 4.15	88.2	4.7	86.62 ± 6.84	86.6	7.9
	200	182.38 ± 4.89	91.2	2.7	178.69 ± 8.36	89.3	4.7
Pear	50	37.56 ± 1.38	75.1	3.7	38.28 ± 1.35	76.6	3.5
	100	79.41 ± 2.85	79.4	3.6	85.37 ± 1.82	85.4	2.1
	200	173.86 ± 11.52	86.9	6.6	193.77 ± 3.94	96.9	2.0
Orange	50	46.63 ± 2.11	93.3	4.5	36.91 ± 1.93	73.8	5.2
	100	99.92 ± 6.06	99.9	6.1	85.36 ± 3.14	85.4	3.7
	200	207.07 ± 5.82	103.5	2.8	180.38 ± 10.15	90.2	5.6

## Data Availability

Data are contained within the article.

## Supplementary Materials

The following supporting information can be downloaded at: https://www.mdpi.com/article/10.3390/foods11203163/s1, Figure S1: Optimization of mAb concentration and phage input in noncompetitive P-ELISA; Figure S2: Matrix effects on competitive P-ELISA. Figure S3: Matrix effects of noncompetitive P-ELISA; Table S1: Phage titer among panning; Table S2: Optimization of mAb concentrations and phage inputs in competitive P-ELISA; Table S3: Comparison of reported ELISAs for IMI.
