# Supplementary material for: Identification and Application of Two Promising Peptide Ligands for the Immunodetection of Imidacloprid Residue
Source: Foods. 2022 Oct 11;11(20):3163. doi: 10.3390/foods11203163 (PMC9602300; doi:10.3390/foods11203163)
Supplement: Supplementary file 1 [file foods-11-03163-s001.zip › foods-1891831-supplementary.pdf]

Supporting Information

Identification and Application of Two Promising Peptide Ligands for the Immunodetection of Imidacloprid Residue

Tianyang You[1,2,†], Yuan Ding[1,2,†], Yue Huang[1,2], Yang Lu[1,2], Minghua Wang[1,2], Xiude Hua[1,2,*]

[1] College of Plant Protection, Nanjing Agricultural University, Nanjing 210095, China

[2] State & Local Joint Engineering Research Center of Green Pesticide Invention and Application, Nanjing 210095, China

[*] Correspondence: huaxiude@njau.edu.cn; Tel./Fax: +86-25-84395479

[†] The authors contributed equally to this work

Table S1. Phage titer among panning.

Rounds	Input (cfu/mL)	Output (cfu/mL)	
		Peptidomimetics	Anti-immunocomplex peptides
1	3.0×10^{11}	6.0×10^{5}	2.2×10^{5}
2	3.0×10^{10}	1.6×10^{8}	4.1×10^{5}
3	3.0×10^{10}	2.0×10^{8}	4.5×10^{7}

Table S2. Optimization of mAb concentrations and phage inputs in competitive P-ELISA.

Clone name	Concentration of antibody (μg/mL)	Input of phage particles (pfu)	OD_{450}
1-9-G	2.5	1.56×10^{8}	1.0148
1-11-H	2.5	7.81×10^{7}	1.2772
1-10-F	2.5	1.95×10^{7}	1.3974
1-12-A	2.5	1.95×10^{7}	1.1289
1-9-H	2.5	3.91×10^{7}	1.2429
1-12-C	1.25	6.25×10^{8}	1.4145
1-10-H	2.5	1.95×10^{7}	1.2585

Table S3. Comparison of reported ELISAs for IMI.

Methods	IC_{50}/SC_{50} (ng/mL)	Detection range (ng/mL)	References
Competitive P-ELISA	0.067	0.024-0.4	[9]
Competitive P-ELISA	96	8.0-906	[27]
Competitive ELISA	35	Not shown	[19]
Competitive ELISA	17.3	5-125	[20]
Competitive ELISA	0.3	0.03-4.0	[21]
Competitive ELISA	1.2	Not shown	[22]
Competitive ELISA	121.6	Not shown	[23]
Competitive ELISA	1.3	Not shown	[24]
Competitive P-ELISA	0.55	0.30-1.00	Present work
Noncompetitive P-ELISA	0.35	0.15-0.8	Present work

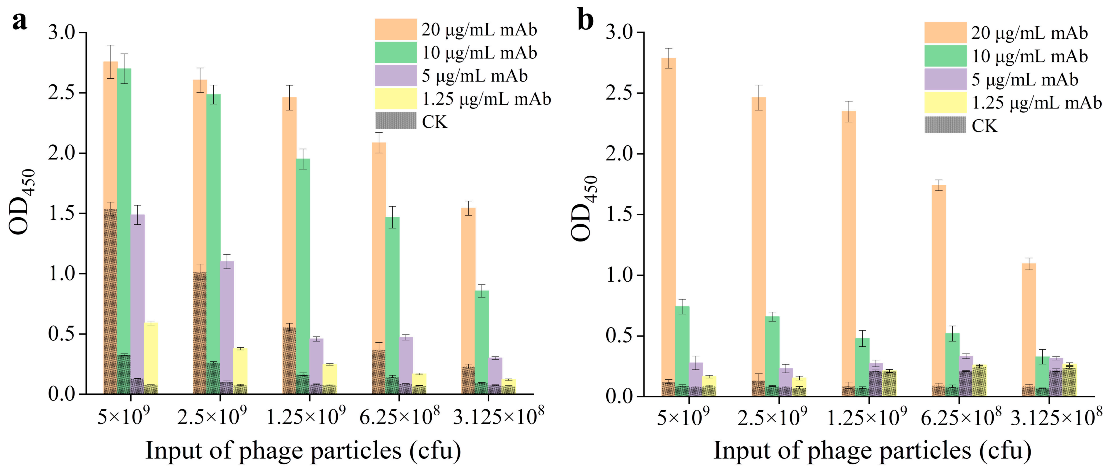

Figure S1. Optimization of mAb concentration and phage input in noncompetitive P-ELISA.

(a) Peptide 2-1-A. (b) Peptide 2-1-H.

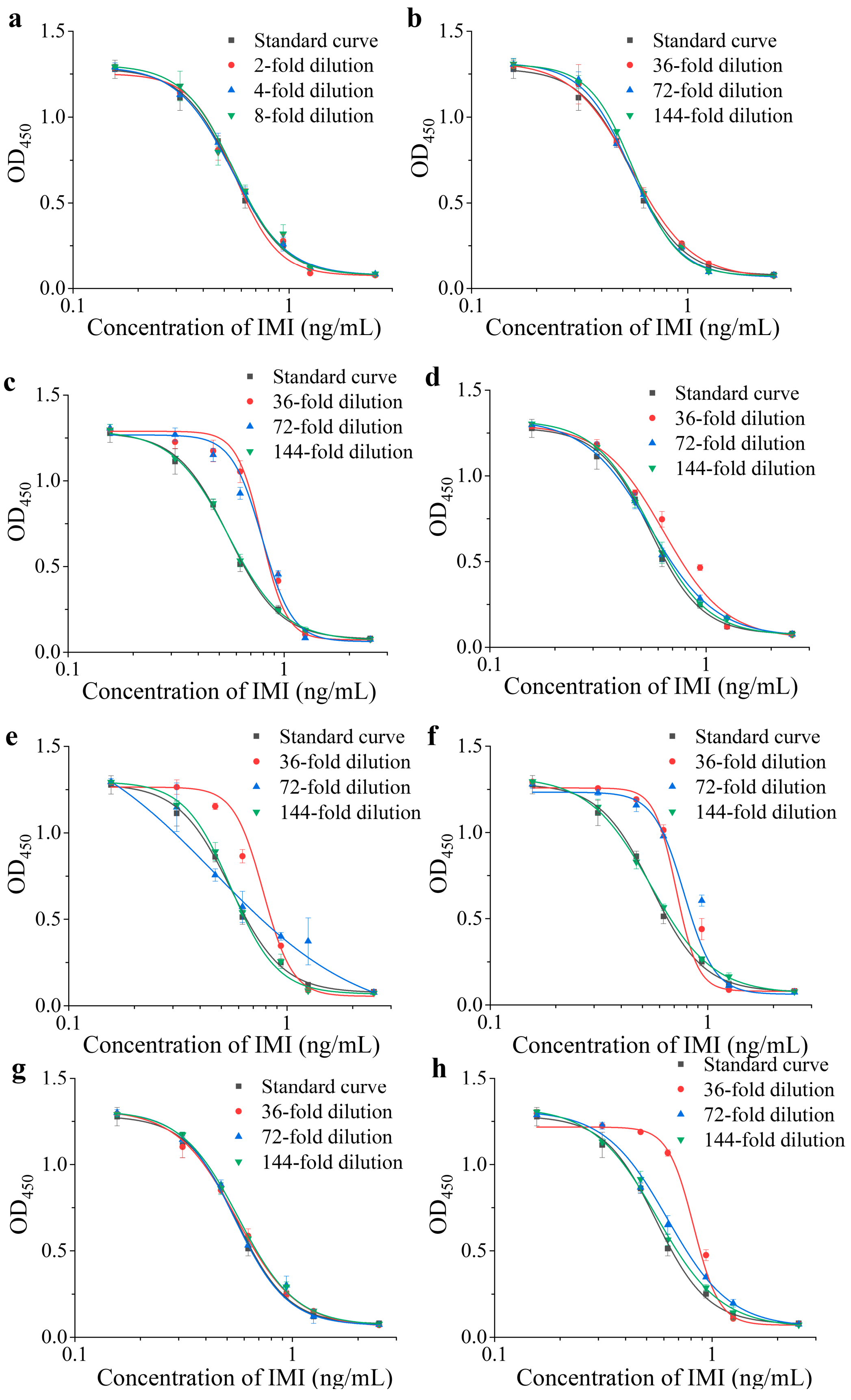

Figure S2. Matrix effects on competitive P-ELISA. (a) Paddy water. (b) soil. (c) wheat. (d) brown rice. (e) cabbage. (f) potato. (g) pear. (h) orange.

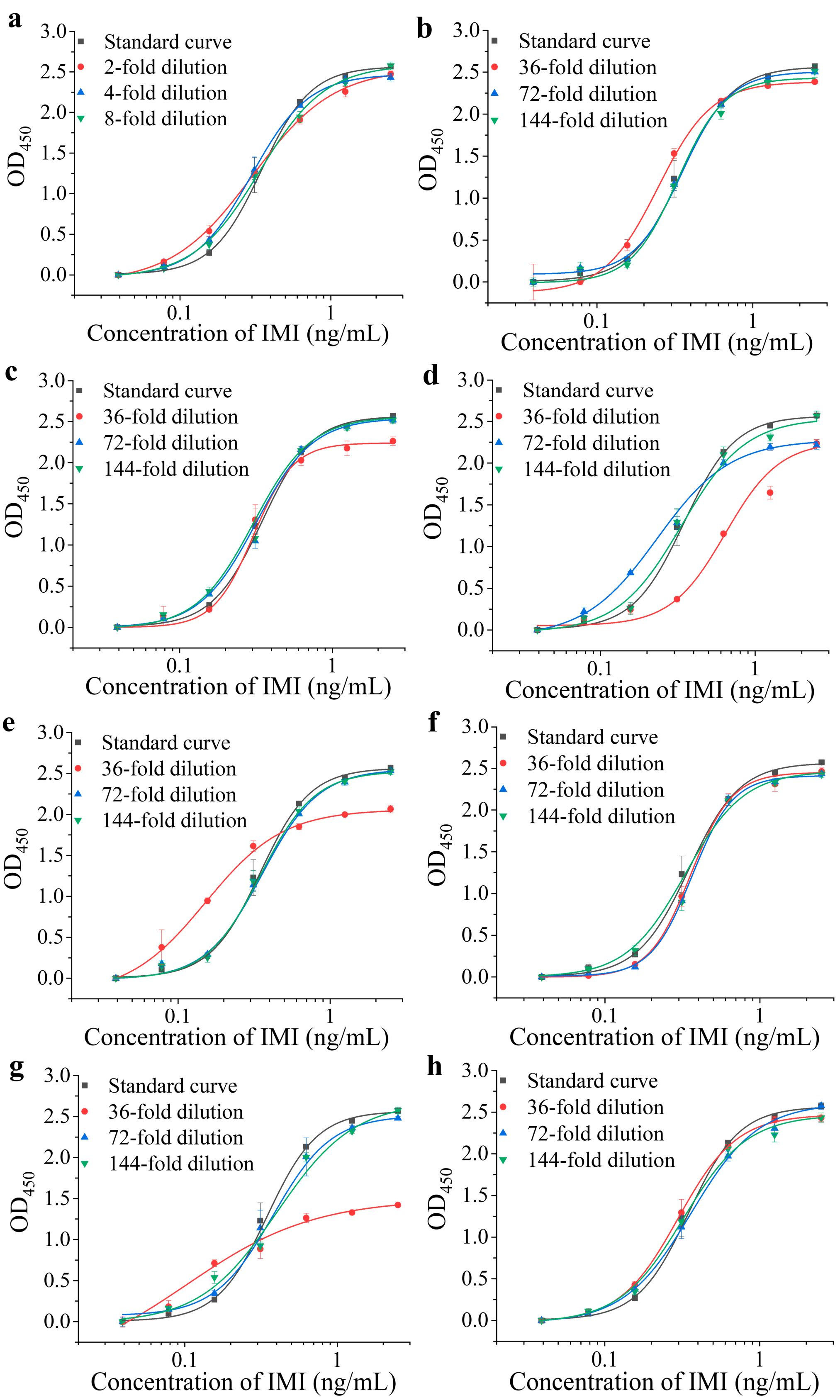

Figure S3. Matrix effects of noncompetitive P-ELISA. (a) Paddy water. (b) soil. (c) wheat. (d) brown rice. (e) cabbage. (f) potato. (g) pear. (h) orange.
